# A Joanna Briggs Institute Framework Approach to Shared Decision Making in End‐of‐Life

**DOI:** 10.1111/hex.70041

**Published:** 2024-10-07

**Authors:** Marta Gil Glaría, María Martín Fernández, Carla Salgado, María José Hernández‐Leal

**Affiliations:** ^1^ Oncology Inpatient Service Clínica Universidad de Navarra Madrid Spain; ^2^ Department of Community, Maternity and Pediatric Nursing, Campus Universitario University of Navarra, School of Nursing Pamplona Spain; ^3^ Faculty of Medicine University of Azuay Cuenca Ecuador; ^4^ PhD Program in Medical Sciences University of La Frontera Temuco Chile; ^5^ Millennium Nucleus on Sociomedicine Santiago Chile; ^6^ IdiSNA Navarra Institute for Health Research Pamplona Spain

**Keywords:** decision aids, end of life, oncology, palliative care, shared decision‐making

## Abstract

**Aim:**

To implement shared decision‐making (SDM) through a patient decision aid (PtDA) for the initiation of palliative care (PC) in end‐of‐life (EOL) cancer patients.

**Methodology:**

A comprehensive Scoping Review was conducted on SDM in PubMed, CINAHL and PsycInfo. An evidence‐based implementation of PtDAs was created using the Joanna Briggs Institute framework, which followed rigorous pillars: (1) context, (2) facilitation and (3) evaluation.

**Results:**

Fifteen studies were identified and categorised into (1) Implementation characteristics and (2) Strategies for implementing SDM in terminally ill cancer patients. SDM should consider the decision‐making location, optimal timing, participants and decision type. Strategies include professional training, PtDAs and implementation programmes. A PtDA implementation protocol in video format for deciding to initiate PC is proposed, following International Patient Decision Aid Standards (IPDAS) and Clinical Practice Guidelines (CPG).

**Conclusions:**

SDM implementation should be guided by evidence‐based methodological models justifying and structuring its execution, especially in complex and interdisciplinary contexts. National or international frameworks facilitate the adoption of health innovations, such as PtDAs, benefiting patients and improving their usage.

**Practice Implications:**

SDM is not just a concept but an important approach to the Care of cancer patients at EOL, enhancing patient satisfaction and improving care quality. The success and sustainability of SDM hinge on the fundamental aspects of staff training, interdisciplinary collaboration and ongoing evaluation. The lack of specific aid in Spanish underscores the immediate need for local development. Further research is needed in this area, as most reviewed studies did not measure SDM effectiveness in diverse hospital settings.

**Patient or Public Contribution:**

This proposal was developed based on the experience and input of the nursing staff from the healthcare service where it is intended to be implemented.

## 1 Introduction

Shared decision‐making (SDM) represents a shift from a paternalistic approach to a collaborative relational model between healthcare professionals and patients, marking a key aspect of person‐centred care (PCC) [1]. SDM, which originated in the 1970s in Western countries [2], aims to shift from a paternalistic paradigm where healthcare professionals provide therapeutic recommendations based on clinical experience and scientific evidence. SDM is an evidence‐based approach that considers both patient preferences and values and offers a participatory and horizontal model between patients and healthcare professionals [2, 3]. Its implementation in various health contexts has demonstrated benefits, including increased treatment adherence, higher satisfaction, reduced patient worries and anxiety and decreased decisional conflict [4, 5]. These benefits are particularly significant in the context of oncology and palliative care (PC), where the decisions made can have a profound impact on the quality of life and end‐of‐life (EOL) experiences of patients [6].

Initially applied in uncertain contexts where the risk‐benefit relationship or evidence did not provide a clear recommendation [7, 8], SDM's newer definitions seek to expand it to situations where a patient's humanity or identity is at stake, such as patients at the EOL [9]. Hence, our objective is to propose an implementation of a patient decision aid (PtDA) regarding the initiation of palliative care (PC) in terminally ill cancer patients, addressing the decision to initiate PC or continue with active therapy. To achieve this, a scoping review was conducted initially to explore strategies facilitating SDM implementation in adult oncology patients at the EOL, along with the recommended characteristics and circumstances for implementation. Subsequently, these results informed a set of recommendations to establish an action plan for SDM in the Oncology Inpatient Ward at an institution in Spain, utilising the Joanna Briggs Institute (JBI) framework [10]. The JBI framework offers a detailed approach for systematic reviews and evidence synthesis, based on three key pillars: context, facilitation and evaluation. It provides clear guidelines for defining research questions, creating protocols, searching for and selecting studies and assessing and synthesising evidence. JBI's quality criteria focus on evaluating the robustness of study design, the risk of bias, data reliability and the relevance of findings to clinical practice [10].

## 2 Literature Review

A scoping review was conducted in March 2023 to explore implementation strategies for SDM in adult oncology patients at the EOL (OSF register Identifier: DOI 10.17605/OSF.IO/XFJ6H). The methodological recommendations from the Cochrane Review Manual were followed during its development [11].

**Figure 1:**
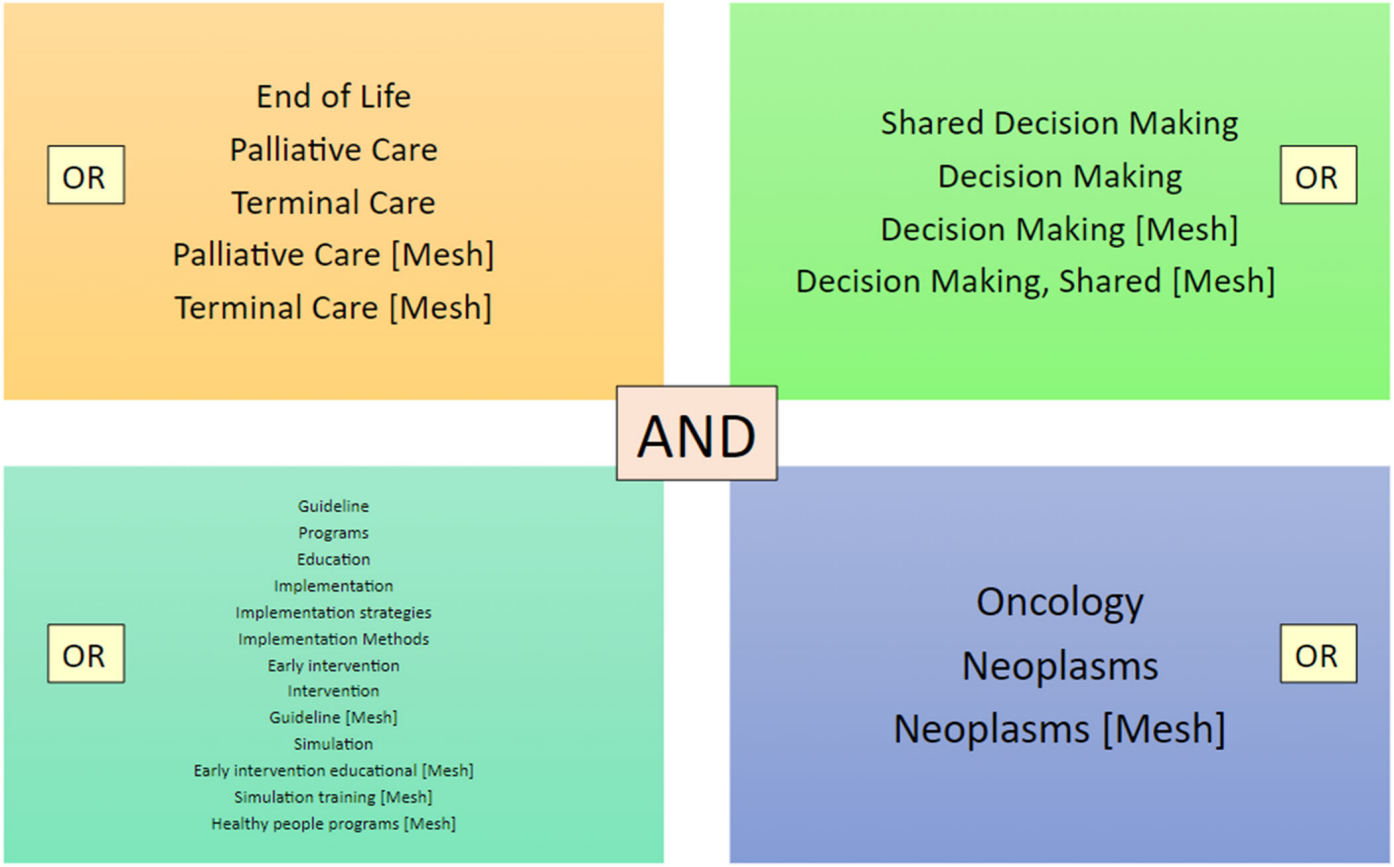
Search strategy.

Literature search was performed across three databases: PubMed, CINAHL and PsycInfo. The terms ‘Implementation’, ‘Shared Decision‐Making’, ‘Oncology’ and ‘Palliative Care’ along with their synonyms, MeSH terms and Thesaurus were used. Their combination was achieved using the boolean operators ‘AND’ and ‘OR’ (Figure 1). Selection criteria are shown in Table 1.

**Table 1:** Selection criteria.

	Inclusion criteria	Exclusion criteria
Types of studies	No restrictions	Not applicable
Participants	Cancer patients at the end of life (EOL) and/or their support network (family, friends, etc.)	Paediatric patients Patients with cognitive impairment
Interventions	Decision‐making developed through negotiation between healthcare professionals and the patient and/or family members	Implementations addressing informed decision‐making
Outcomes	Strategies, interventions, guidelines, programmes, training to incorporate SDM	Not applicable
Limitations	Languages: English, French and Spanish	Not applicable

Abbreviation: SDM, shared decision‐making.

**Figure 2:**
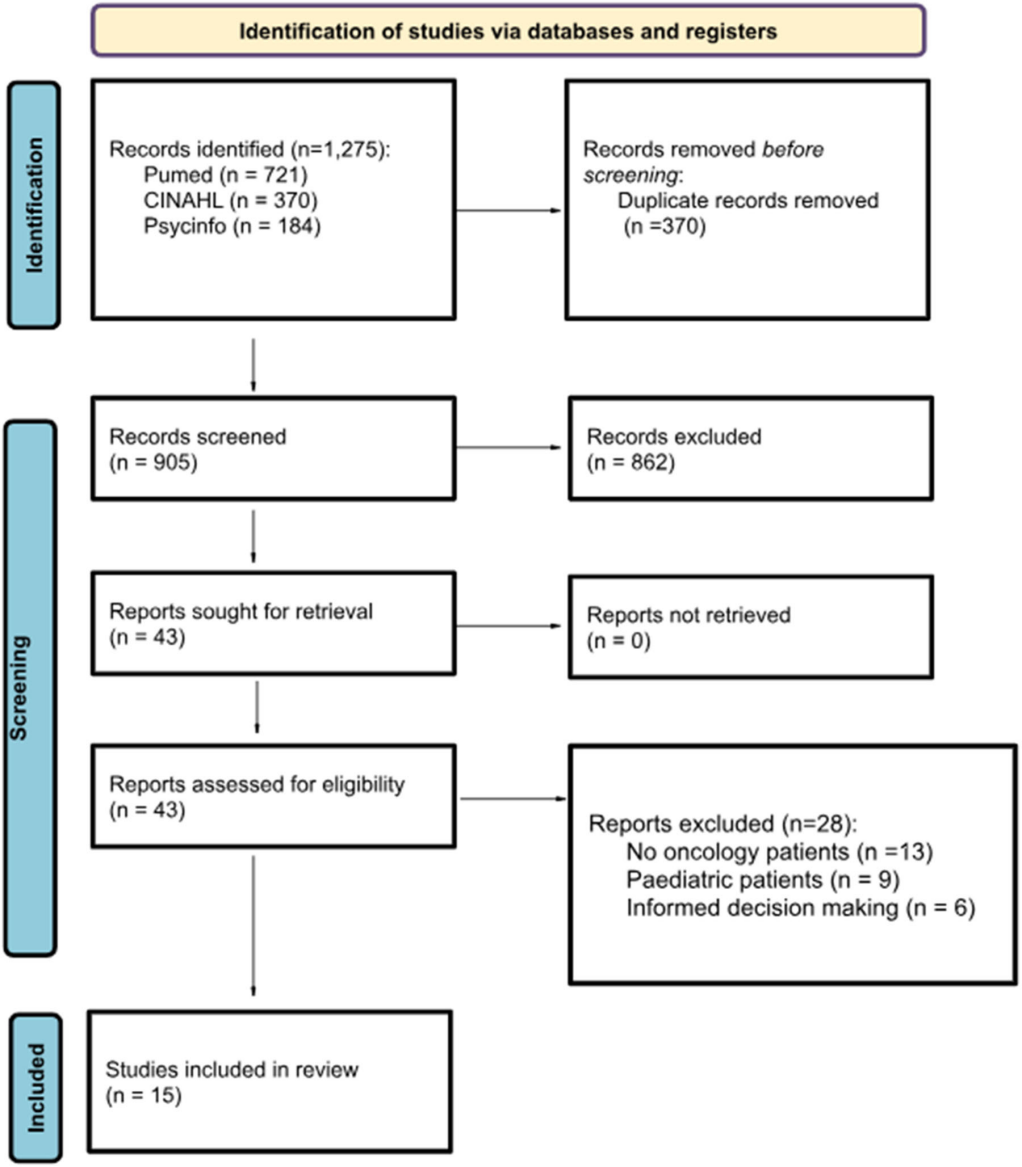
Flowchart scoping review [13].

After eliminating duplicates, the titles and abstracts were screened independently by two researchers (M.G.G. and M.J.H.‐L.). To assess the inter‐rater reliability, two researchers independently screened 20% of the articles. The resulting kappa statistic was 0.81, indicating a high level of agreement [12]. In case of disagreement, the researchers discussed the case until they reached a consensus. Subsequently, one researcher made the article selection through full‐text reading. A total of 15 articles were selected for final analysis (Figure 2).

The geographical distribution included the Netherlands (*n* = 6), the USA (*n* = 6), followed by Taiwan (*n* = 1), France (*n* = 1) and Australia (*n* = 1). Based on JBI quality criteria, seven studies were of high quality (the compliance score was equal to or greater than 70%), seven studies fell in the moderate quality category (score between 50% and 70%) and one article was categorised as low quality (score less than 50%). Regarding study designs, we found five randomised clinical trials (RCT), five RCT protocols, two quasi‐experimental studies, one cross‐sectional study, one narrative review and one qualitative study (Table 2).

**Table 2:** Characteristics of the results in the scoping review.

Authors, country	Design study	Aim(s)	Sample characteristics	Implementation characteristics	Strategies for the implementation of SDM	JBI quality
Henselmans et al. [14], the Netherlands	RCT	The CHOICE (Choosing Treatment Together in Cancer at the End of Life) trial aims to add high‐quality evidence on the effectiveness of communication interventions by examining both the separate and combined effect of an SDM training for medical oncologists and a patient communication aid (PCA) on observed SDM about palliative systemic treatment.	24 oncologists and 192 patients diagnosed with metastasis or inoperable tumours.	PROFESSIONAL TRAINING Location: Hospital Participants: Oncologists with patients Timing: not reported Decision type: Treatment	Training modality: CHOICE (Choosing Treatment Together in Cancer at End of Life). Measurement instruments: OPTION 12, four SDM, nine‐item SDM Questionnaire, five‐item Patient Satisfaction Questionnaire, 16‐item Decisional Conflict Scale PtDA: PCA Outcome: Oncologists were trained in SDM through the CHOICE programme. Participation data: Oncologist training improved SDM (*p* < 0.001); PCA did not. The combination was equal to the effect of training alone. Training improved patient‐reported SDM; PCA did not; the combination did not enhance the training effect. The condition did not affect patient satisfaction (*p* = 0.97) or oncologist satisfaction (*p* = 0.26) with communication. The condition did not affect patient decision regret (*p* = 0.11) or patient quality of life (*p* = 0.87). Consultations lasted 5 min longer for oncologists trained in unassisted patient consultations (*p* = 0.01).	84.6%
Bos‐van den Hoek et al. [15], the Netherlands	RCT with a pre‐posttest group	Examine the effects of a blended online learning for oncologists about SDM in palliative oncological care and to compare this blended format with a more extensive, fully in‐person face‐to‐face training format.	15 oncologists.	PROFESSIONAL TRAINING Location: Hospital Participants: Oncologists with patients Timing: Not reported Decision type: Palliative treatment	Training modality: Professional training through e‐learning. Measurement instruments: OPTION 12, four SDM, PSQ. PtDA: Pocket card with the four steps of SDM and example phrases. Outcome: Online training to implement SDM. Participation data: Oncologists demonstrated significantly higher levels of SDM after blended online learning, as measured by OPTION 12 (*p* < 0.001) and four SDM (*p* < 0.001). Oncologists' satisfaction with the conversation (*p* < 0.001) improved.	53.8%
Mohan et al. [16], USA	Quasi‐experimental	Describe communication practices of physicians making treatment decisions for unstable critically ill patients with end‐stage cancer, using a shared decision‐making model.	13 emergency department physicians, eight intensivists and six general practitioners.	PROFESSIONAL TRAINING Location: Hospital Participants: Physicians with patients Timing: Critical unstable situations Decision type: Treatment (ICU or palliative care)	Training modality: Not reported. Measurement Instruments: Behaviours, communication skills. PtDA: Not applicable. Outcome: Simulation to assess how physicians make decisions for terminal cancer patients in a critical situation using the SDM. Participation data: Predictors of communication skills: Physician characteristics, years since graduation, general and associated specialties. Behaviour ‘Obtaining and responding to preferences’ *p* = 0.03, ‘affirming patient decisions’ *p* = 0.01 were negatively associated with years since graduation. Predictors of treatment decisions: Intensivists and emergency physicians more likely to admit to the ICU *p* = 0.21. Obtaining the predicted palliation patient objective skill score *p* = 0.04.	66.7%
Van Veenendaal et al. [17], the Netherlands	RCT, protocol	Address the effectiveness of an individual SDM training programme using the concept of deliberate practice.	Implementation phase study, intended to be conducted across 12 hospitals.	PROFESSIONAL TRAINING Location: Hospital Participants: Oncologists with their patients Timing: During the clinical encounter Decision type: Treatment	Training modality: e‐learning, personal learning and in‐person coaching. Measurement instruments: OPTION‐5, SCPS, SDM‐Q‐9. PtDA: Not applicable. Outcome: Training for oncologists to implement SDM Participation data: Not applicable.	69.2%
Oostendorp et al. [18], the Netherlands	RCT phase II	Evaluate any harmful effects of the DAs as compared with usual care, regarding patients' well‐being and specifically anxiety.	128 patients with colorectal or breast cancer and 20 nurses.	DECISION AIDS: Location: Hospital Timing: Not reported Participants: Nurses and oncologists with patients Decision type: Second‐line chemotherapy treatment	Information modality: PtDA booklets with risks and benefits of treatment are presented, including a life expectancy illustration. Measurement instruments: HADS for anxiety and HRQoL PtDA: Colorectal and breast cancer patients, unspecified type. Outcome: Oncologists completed an inclusion form, and nurses completed a questionnaire about the interview with PtDA. Patients filled out an initial questionnaire at enrolment and two follow‐up questionnaires one week and eight weeks after receiving treatment‐related information. Participation data: Decision‐related measures (the intervention group had stronger treatment preferences, *p* = 0.03). Treatment attitudes (both groups equally satisfied with their treatment and its consequences).	61.5%
Dharmarajan et al. [19], USA	Quasi experimental	Build a video decision aid for hospitalised patients with advanced cancer referred for PRT and prospectively test its efficacy in reducing decisional uncertainty, improving knowledge, increasing treatment readiness and readiness for palliative care consultation and its acceptability among patients.	40 patients with advanced cancer: 23% prostate cancer, 18% lung cancer and 15% ovarian cancer.	DECISION AIDS Location: Hospital and outpatient clinic Participants: Not reported Timing: Hospitalised patients about to receive PRT Decision type: Receiving PRT	Information modality: Video decision aids: (1) The radiation simulation process, (2) What to expect during treatment, (3) Side effects, (4) The purpose of palliative care. Patients then answer questions within 24 h after viewing. Measurement instrument: Three‐item Decisional Conflict Scale. PtDA: Video. Outcome: Through the video, patients are educated to make decisions about palliative care and receiving PRT when hospitalised. Participation data: Patients were more confident in their decision (*p* = 0.02). The effect was greater among patients without prior PRT (*p* = 0.02), with no difference with prior PRT (*p* = 0.28). Willingness to accept PRT improved (*p* = 0.04) and patients felt more prepared for PRT after the video. Mean knowledge increased from 60.4 to 88.3, *p* < 0.001.	77.7%
Agarwal and Epstein [20], USA	Narrative review	Emphasise the undeniable value, current challenges and recent improvements in supporting optimal ACP	No reported.	IMPLEMENTATION PROGRAMMES Location: Not reported Participants: Medical team with patients and families Timing: Not reported Decision type: Treatment, palliative care	Information modality: Not reported. Measurement POLST. DAs: Not applicable Outcome: Patient treatment expectations and understanding of the illness, uncertainty of prognosis with evolving cancer therapies, optimal timing of SDM discussions, barriers in doctor–patient communication, heterogeneity in quality, content, approach and documentation of SDM discussions. Participation data: Improvement Strategies of Integration of primary palliative care and nurse‐led interventions, patient‐centred and value‐focused SDM models, including the use of patient‐reported outcomes, participation in interactive SDM discussions, use of technology to enhance communication, standardisation of SDM principles and documentation. Empathetic and honest conversations among patients, caregivers and healthcare professionals can prevent aggressive and unwanted end‐of‐life care.	18%
Dionne‐Odom et al. [21], USA	RCT	Assess the feasibility, acceptability and potential efficacy of individual intervention components of CASCADE (CAre Supporters Coached to be Adept DEcision Partners), an early telehealth, palliative care coach‐led decision support training intervention for caregivers.	46 patients and 46 family members.	IMPLEMENTATION PROGRAMMES Location: Tertiary academic medical centre Participants: Oncologists with patients and family members Timing: Newly diagnosed with advanced cancer Decision type: Treatment, roles of values when deciding with and for others and supporting the completion of advance directives and being a durable power of attorney for healthcare	Information modality: Not reported. Measurement instrument: Rini Decision Influence Scale. PtDA: Not applicable. Outcome: CASCADE: (1) Psychoeducation for effective decision support (one or three sessions for deciding on treatment), (2) communication training for decision support and (3) Ottawa Decision Guide training (one session reviewing the four steps of the guide and how they can be used with patients). Patients were only informed and did not receive an intervention. This intervention was conducted on the oncologists. Family caregivers were paired with a trained palliative care coach who scheduled and facilitated a series of one to five weekly phone sessions lasting 20 to 30 min. Participation data: Regarding caregiver distress and depressive symptoms, results suggest that the most beneficial components were training in communication for decision support (*d* = −0.49–0.25). However, for caregiver anxiety, the only beneficial component was training in communication for decision support (*d *= −0.26). Regarding perceived positive decision influence by the patient, the component resulting in the greatest effect was communication training for decision support (*d* = 0.62); however, the psychoeducation component for effective decision support also resulted in a relevant albeit lower magnitude effect (*d* = 0.33).	69%
Fritz et al. [22], the Netherlands	Qualitative study utilising focus groups with healthcare professionals and semi‐structured interviews with patients and their representatives.	Develop an ACP programme specifically for glioblastoma patients. Evaluated topics that are relevant for patients and their proxies and facilitators and barriers to participating in an ACP programme.	10 healthcare professionals (two neuro‐oncologists, one neuro‐oncology nurse, one oncology nurse, two neuro‐oncology radiation therapy oncologists, two palliative care nurses, one general practitioner, one home care nurse, one end‐of‐life researcher). 13 patients with glioblastoma and their family members, and six family members of deceased patients.	IMPLEMENTATION PROGRAMMES Location: Not reported Participants: Medical team with patients and family members Timing: After diagnosis or chemotherapy Decision type: Treatment: ACP	Information modality: Not reported. Measurement instrument: Not reported. PtDA: Not applicable. Outcome: Topics addressed in both the focus group and interviews include (1) current situation, (2) concerns and fears, (3) treatment options and (4) preferred location for care and death. Participation data: Not applicable.	80%
Hoerger et al. [23], USA	RCT, protocol	Help doctors, patients with advanced cancer and caregivers communicate more effectively about topics that may influence decision making.	40 oncologists and 400 patients with advanced cancer, each accompanied by a family member.	IMPLEMENTATION PROGRAMMES Location: Not reported Participants: Oncologists with patients and family members Timing: In patients with incurable cancer before a critical situation, anticipating informational needs and strengthening the physician–patient relationship Decision type: Treatment choice, symptom management, transition to palliative care	Information modality: VOICE (Values and Options in Cancer Care): Oncologist training through DVD + guidelines. Patient training through a booklet. Measurement instruments: APPC, PTCC, CPS, SPI and Actual Decision Role. PtDA: DVD + Communication Guide ‘reminder’ card for oncologists. QPL, My Cancer Care booklet for patients and family members. Outcome: VOICE: Phase 1 involves preparing oncologists in SDM (DVD of 15 min, communication guide ‘reminder’ card encouraging discussions on topics such as prognosis and symptoms found in the QPL, role‐playing exercise). Patients and caregivers receive training and intervention, and results are assessed (1 h) after completing a survey (during training, they fill out a QPL organised by the My Cancer Care booklet). Participation data: Not applicable.	76.9%
Korfage et al. [24], USA	ACTION‐multicentre RCT	evaluate the effects of a complex ACP intervention on the quality of life, operationalised as emotional functioning and symptoms of patients with advanced lung or colorectal cancer. Coping, patient satisfaction, SDM, patient involvement in decision making, AD inclusion in hospital files and use of hospital care.	1117 patients with lung or colorectal cancer, caregivers and 39 healthcare professionals (especially nurses).	IMPLEMENTATION PROGRAMMES Location: Hospital or at home Timing: Not reported Participants: Nurses trained in ACTION RC with patients and family members Decision type: Treatment, CPR, future care goals	Information modality: Informational brochures ‐Information on CPR, artificial ventilation and artificial feeding‐. Measurement instruments: Satisfaction with care (EORTC IN‐PATSAT), satisfaction with the intervention (9 items created by the study), SDM; APECC. PtDA: Informational brochures. Outcome: ACTION RC Programme ‐ SDM: (1) Facilitated structured SDM conversations, (2) Preference form: My preference form, (3) informational brochures. Participation data: No differences were observed in coping, satisfaction with care, patient involvement in decision‐making or shared decision‐making. The intervention group more frequently utilised specialised palliative care.	76,9%
Michael et al. [25], Australia	RCT, protocol	Evaluate the potential utility of a video decision support tool (VDST) that models value‐based ACP discussions between cancer patients and their nominated caregivers to enable patients and families to achieve shared‐decisions when completing ACP's.	86 patients with incurable cancer and 112 family members.	IMPLEMENTATION PROGRAMMES Location: Hospital Timing: Not reported Participants: Oncologist with patients and family members Decision type: Pharmacological treatment and its effects on end‐of‐life quality of life	Information modality: Vignettes presented to patients and caregivers in interviews and focus groups to gather perspectives on SDM. Measurement instruments: Attitudes towards SDM; questionnaire measured on a 10‐point Likert scale. Decision‐making congruence: CCAT‐P and CCAT‐F. Decision‐making readiness: Decision‐making preparedness scale. PtDA: VSDT. Outcome: Vignettes facilitate communication between patients and caregivers. Participation data: Not applicable.	61.5%
Rietjens et al. [26], the Netherlands	Multicentre‐cluster‐RCT, protocol	To evaluate the effect of the ACP RC programme on patients' quality of life and symptoms, to what extent care is received according to patients’ preferences, evaluation of the quality of the decision‐making process, how they cope with their illness, patient satisfaction, quality of end‐of‐life care and cost‐effectiveness	1360 patients with advanced lung cancer (*n* = 680) or colorectal cancer (*n* = 680).	IMPLEMENTATION PROGRAMMES Location: Hospital Timing: Not reported Participants: Nurses with the patient and family members Decision type: Pharmacological treatment and care in critical situations, such as cardio‐pulmonary resuscitation	Information modality: ACTION: An interview is conducted (45–60 min), one or two sessions per patient. Subsequently: (1) My preference form (questionnaire on quality of life, decision‐making process, coping and care satisfaction), (2) Medical record review, (3) Study of recorded ACP sessions. Measurement instrument: Not reported PtDA: Not applicable Outcomes: Nurses are trained in the ACTION programme for SDM and then they implement it with patients. The patient and personal representative will engage in a facilitated SDM conversation following the ACTION programme. Nurses will assist the patient in documenting their preferences, including appointing a personal representative. Participation data: Not applicable.	76,9%
Tang et al. [27], Taiwan	RCT	Examine an interactive ACP intervention tailored to participants' readiness to engage in ACP while monitoring/ensuring high treatment fidelity.	430 patients with terminal cancer.	IMPLEMENTATION PROGRAMMES Location: Not reported Timing: Not reported Participants: Not reported Decision type: Choosing between LST and improving QoL	Information modality: During an interview, participants are trained and provided with videos and informational pamphlets. Measurement instruments: Preference for LST: adapted interview protocol. QoL: 13‐item MQoL. Benefits and hazards of treatment. PtDA: Pamphlets and educational video aid. Outcome: The five components of the ACP intervention: (1) participant assessments, (2) interventions to engage in ACP, (3) discussions on end‐of‐life care between physician and patient, (4) pamphlet and educational video aid to facilitate understanding of ACP and LST at end of life, (5) psychological support. Participation data: The ACP intervention did not facilitate concordance between preferred and received LST, nor did it impact the quality of life during the dying process**.**	84.6%
Tricou et al. [28], France	Cross‐sectional study	Describe the decision‐making process and the DCPs of patients with advanced cancer receiving palliative care	200 patients with advanced cancer referred to palliative care.	IMPLEMENTATION PROGRAMMES Location: Not reported Timing: Not reported Participants: Oncologists with their patients Decision type: Not reported	Information modality: Four questionnaires (1) Demographic characteristics, (2) Preferences (passive, active or shared decision‐making), (3) Patient satisfaction with decisions and care, (4) Patient's level of understanding of their illness, treatment and prognosis. Measurement instruments: Control preference scale (Degner and Sloan), Decision satisfaction scale and level of understanding of their disease, treatment and prognosis. PtDA: Not applicable Outcome: Not applicable; there is no intervention. The study assesses the SDM process through questionnaires. Participation data: (1) Age, where younger age is associated with more active SDM (*p* = 0.003), (2) Education, with higher education associated with more active or SDM (*p* < 0.001), (3) Employment status, where working patients have more active or SDM (*p* = 0.046).	50%

Abbreviations: ACP, advanced care planning; AD, advance directrices; APECC, assessment of patients' experience of cancer care; APPC, The Active Patient Participation Coding; CASCADE, CAre Supporters Coached to be Adept DEcision Partners; CHOICE, Choosing Treatment Together in Cancer at End of Life; CPR, cardio‐pulmonar resuscitation; CPS, control preferences scale; DCP, decision control preference; EORTC IN‐PATSAT, European Organisation for Research and Treatment of Cancer; ICU, intensive care unit; LST, life‐sustaining treatment; MQoL, McGill QoL Questionnaire; OPTION 12, Observing Patient Involvement scale; PC, palliative care; PCA, patient communication aid; PtDAs, decision aids; POLST, Physician Orders for Life‐Sustaining Treatment; PRT, palliative radiation therapy; PTCC, The Prognostic and Treatment Choices; QoL, quality of life; QPL, Question Prompt List; RC, respecting choices; RCT, random clinical trial; SDM, shared decision‐making; SPIs, standardised patients instructors; VSDT, video decision support tool.

### 2.1 Setting

All studies indicated that the preferred setting for SDM implementation is the hospital, as it is where patients spend the most time making decisions collaboratively with healthcare professionals [14, 15, 16, 17, 18, 19, 21, 24, 25, 26]. However, two articles mentioned that it could also be conducted at home [19, 24]. They also noted that the timing of implementation should anticipate the patient's information needs, that is, before a critical situation, after diagnosis and/or during the clinical encounter [17, 21, 22, 23]. Dharmarajan et al. [12] and Fritz et al. [22] propose specific moments, such as before receiving chemotherapy or radiotherapy (RDT).

### 2.2 Patients' Conditions

The majority of patients were suffering from glioblastoma [22], lung and colorectal cancer [24, 26], prostate and ovarian cancer [19] and breast cancer [18]. They reported that decisions are predominantly made between patients, physicians and nurses.

### 2.3 Intervention Strategies

#### 2.3.1 Training for Healthcare Professionals

Both physicians and nurses are crucial for conducting SDM effectively with patients and their families [14, 15, 16, 17]. Hoek et al. [15] and Van Veenendaal et al. [17] have developed e‐learning‐based training sessions, lasting 45–60 min, involving video visualisation of the four SDM steps: (1) creating awareness of options, (2) discussing options with their pros and cons, (3) exploring patient values and (4) agreeing on a decision that aligns with patient preferences. Additionally, Van Veenendaal et al. [17] added self‐assessment, self‐learning questions and a 15–30 min in‐person coaching session where professionals discuss feedback.

Another training approach includes communication simulations for physicians on treatment decision‐making for critically unstable patients [16]. On the other hand, Hoerger et al. [23] developed a protocol for the Values and Options in Cancer Care (VOICE) programme. In this programme, oncologists received SDM training through a 15‐min video and ‘reminder’ cards of the communication guide encouraging discussions about prognosis and symptoms listed in the Question Prompt List (QPL).

The Choosing Treatment Together in Cancer at End of Life (CHOICE) training involved 10 h for oncologists and was based on SDM with four steps: (1) an SDM agenda, (2) options and pros and cons, (3) patient values and preferences and (4) deferring a decision. Lastly, a pocket card with the four SDM steps, examples and phrases was provided [14].

The evaluation of the training programmes utilised scales such as the Observing Patient Involvement Scale (OPTION‐12), four‐SDM [14, 15], nine‐item‐SDM [14, 17] and Control Preference Scale (CPS) [16].

#### 2.3.2 Decision Aids

Their aim is to facilitate understanding of therapeutic options, including their risks and benefits, regarding Cardiopulmonary reanimation, artificial ventilation and artificial feeding [24, 27]. They also cover key concepts of PC (palliative care) [19, 27], RDT (radiotherapy) [19], as well as guide SDM [18, 25]. PtDAs can take the form of videos [19, 23, 25, 27] and informational brochures [24, 27].

Dharmarajan et al. [19] have developed a programme that includes a video to educate patients on decision‐making about PC and receiving RDT when hospitalised. The results reported that patients feel more confident in their decision, as measured by the Decisional Conflict Scale (DCS) and improved their knowledge. Additionally, Michael et al. [25] plan to implement the Cancer Communication Assessment Tool for Patients and Families (CCAT‐P and CCAT‐F) in a future study to measure congruence in decision‐making and a scale developed by the authors to measure preparedness for decision‐making.

#### 2.3.3 Implementation Programmes

These consist of multiphase strategies to initiate SDM. The ACTION programme [24] involves 45–60 min interviews with patients conducted by certified professionals following a conversation guide to explore their understanding of the illness, reflect on their goals, values and beliefs and discuss their treatment and care preferences. Patients also complete the My Preference Form questionnaire regarding quality of life, the decision‐making process, coping with the illness and satisfaction with care. Educational brochures on CPR, artificial ventilation and feeding were provided when necessary [24, 26]. To assess satisfaction, the European Organization for Research and Treatment of Cancer (EORTC IN‐PATSAT), Assessment of Patients' Experience of Cancer Care (APECC) and a four‐item questionnaire created by the study were used. Evaluation of results took place at 11–12 weeks and 19–20 weeks after receiving the intervention. No differences were found post‐implementation in coping, satisfaction with care, patient participation in decision‐making or SDM. However, it was reported that patients attended palliative care more frequently after the intervention [24].

Second, the VOICE protocol by Hoerger et al. [23] involves coaching and completing a questionnaire by patients and caregivers, organised by the booklet My Cancer Care. To measure results, they used the Active Patient Participation Coding (APPC), Prognostic and Treatment Choices (PTCC), CPS and Actual Decision Role questionnaires. As a protocol, associated results could not be reported.

Third, the CAre Supporters Coached to be Adept DEcision Partners (CASCADE) programme was developed based on the Social Support Efficacy Theory and the Ottawa Decision Support Framework. It included psycho‐education in one or three sessions addressing decision‐making tips, values when deciding with and for others and supporting anticipated needs. Caregivers were trained by a palliative care coach in 20–30 min sessions and a monthly follow‐up call [21]. The Rini Decision Influence Scale was used to measure positive decision influence, determining that communication support training had a greater effect than psycho‐education [21].

Lastly, Tang et al. [27] developed a programme combining training through videos and informational brochures. In this case, the evaluation scales used to measure results were not specified, but the intervention did not facilitate alignment between preferred and received EOL care preferences.

## 3 Implementation of a PtDAs to Initiate Palliative Care

**Figure 3:**
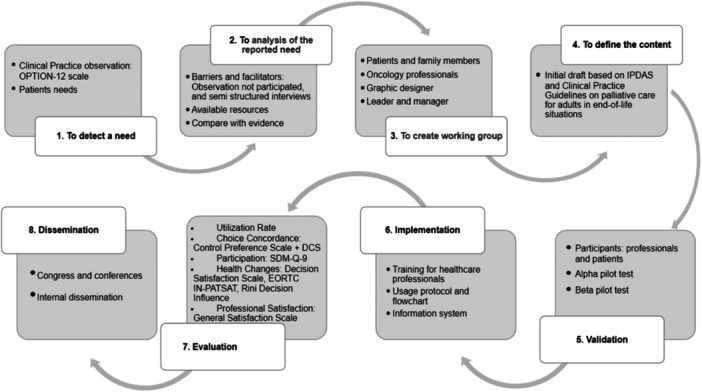
Implementation phases of the decision aid based on the Joanna Briggs Institute framework.

Our evidence‐based implementation proposal is characterised by providing the necessary phases and describing the steps to improve care actions into clinical practice within health services. Consequently, we used the JBI framework Implementation Model [10] as a reference to propose a course of action for implementing a PtDA for patients at the end of life (Figure 3).

The proposal involves implementing a PtDA in video format, utilising IPDAS criteria [29] as a standard and following the CPG on adult palliative care in EOL situations [30]. Our institution will implement this aid in collaboration with healthcare professionals for EOL cancer patients who might be making decisions without adequate informational support.

### 3.1 Context Analysis

The oncology team, often facing critical situations with EOL cancer patients, experiences limited time to learn about patients' preferences for EOL care [31]. Therefore, our goal is to introduce SDM when deciding between invasive care or PC. These decisions are relevant for both the patient and the family, as preparing for this final moment in the company of healthcare professionals is a milestone in people's lives. Patients and their families can find comfort in knowing that their final wishes are respected [32]. Our Scoping Review identified seven PtDAs for EOL patients. However, they have yet to be developed for Spain and do not focus specifically on oncology patients [33].

When developing a PtDA, it is necessary to consider the context and available professionals and have a detailed understanding of the institution. In this case, we will plan it for the oncology hospitalisation unit. Specifically, the principal investigator (PI), the PC team, the oncology team, patients and family members and a graphic designer will participate in developing the PtDA. The research team will ensure the quality and consistency of the PtDA. Second, a management team will participate, including the unit supervisor, the oncology and PC unit medical director and the nursing director. In our setting, the PC team will be responsible for implementing the PtDA in practice due to their proximity to the patient and access to the hospitalisation unit. This team consists of a nurse and a doctor.

After the initial planning, we will embark on developing the content of the PtDA. This process will be thorough, considering recommendations from the CPG on adult PC in EOL situations [30]. We will share this information with healthcare professionals, patients and family members, who will assess the relevance, clarity and appropriateness of the language used in the proposed topics (Agència de Qualitat I Avaluació Sabarrnitàries de Catalunya, n.d.). We will conduct two online meetings with all stakeholders to reach a consensus.

Once the content is defined, we will develop the video PtDA using Dharmarajan et al. [19] as a reference. The PtDA will have a maximum duration of 10 min. The video will be divided into three phases: (1) characteristics of PC (What PC is and implications for the patient and family), (2) situation assessment (Comparison phase between PC and active treatment regarding topics such as risks and benefits, costs, emotional burden, caregiver role, disease progression, lifestyle changes) and (3) open‐ended questions to introduce dialogue based on patient preferences about the discussed topics.

### 3.2 Facilitation of Change

First, assessing the resources available to carry out the change is necessary; this includes human resources, who should be motivated and leadership‐capable professionals. The institution's support in producing the video and technical support for audiovisual needs will be crucial. In terms of financial resources, the institution should subsidise the project. Regarding material resources, the implementation of the PtDA will take place in the hospitalisation unit, requiring no additional space. However, the institute will be responsible for acquiring equipment such as tablets to view the video and to complete evaluations. Also, a software programme such as REDCap [34] for electronic data recording is necessary.

It is essential to identify context‐specific barriers and facilitators for the implementation. The five most reported barriers are inapplicability of the clinical situation to implement PtDAs due to patient and clinical situation characteristics; disagreement with asking the patient about their preferred role in decision‐making; perception that the process and provided DAs are not modifiable; time pressure for both training and implementing the intervention and lack of familiarity with the process and provided PtDAs [35, 36, 37]. Regarding facilitators, six main factors include clarifying values, motivation of health professionals for the results, compatibility with PCC or evidence‐based care, ease of understanding and implementation and shared responsibility with the patient [34, 36]. According to this information, some factors considered as barriers can also act as facilitators. Therefore, it would be interesting to identify them within the institution. Nonparticipant observation and semi‐structured interviews with key participants, decision‐makers and patients are suggested [38]. The goal is to identify key informants and assess how decisions are currently being made in the institution, using the OPTION‐12 scale in the PC team, the nursing team on the hospitalisation unit and the oncology team.

Once the context is analysed, professionals using the PtDA will participate in the Ottawa Hospital's educational programme on SDM. The aim is to improve their skills in engagement, motivation and knowledge related to PtDA implementation [39]. This training focuses on skill development, including considering different options and their pros and cons, understanding and using evidence‐based medical information, preparing to discuss decisions and implementing the options chosen by patients. The PI will be in charge of carrying out the course in person or online. To ensure the quality of training and SDM, we will use a script called the ‘Ottawa Personal Decision Guide’ [35, 40], which provides decision‐making advice and an example video of a case related to SDM [41]; at the end of the course, we will use the Decision Support Analysis Tool (DSAT‐10) to assess the quality of participant's communication skills. Additionally, researchers will conduct a simulation scenario to practise skills and provide feedback to healthcare professionals regarding patient engagement in this specific context [42] as the oncology and PC departments are crucial to ensure the success of implementation. Every time the oncology service requests a PC evaluation for their patients, computer messages will appear to remind the PC team to use the PtDA. Additionally, researchers will create a user‐friendly implementation protocol, including a workflow to clarify the process and how to use the PtDA. The use of the aid could optimise procedure costs and healthcare resources, as some studies have shown that in the context of SDM, patients tend to opt for less invasive options, which could generate significant savings in terms of procedures and costs [43]. However, this effect will need to be evaluated through economic analysis studies to confirm it.

### 3.3 Evaluation of Process and Outcome

Successfully integrating evaluation and implementation strategies is fundamental to ensuring the implementation protocol's sustainability over time. In this context, the process evaluation, a key component, is designed to assess the PtDA's functionality and acceptability. It provides valuable insights into further development and implementation, thereby playing a crucial role in the internal and external validation of the proposed implementation protocol.

First, researchers will conduct a process evaluation through an alpha pilot of the PtDA, involving external stakeholders such as the palliative care team, the oncology team and patients and family members. This multidimensional approach allows for determining the relevance of the content and collecting valuable feedback for adjustments. Subsequently, researchers will conduct a beta pilot, incorporating corrections derived from the alpha pilot, to create a preliminary version of the PtDA subject to validation. Specific quality criteria, such as those established by International IPDAS, will be employed to ensure the robustness of the final version to be implemented in practice. For the beta pilot, we will perform a randomised clinical trial (RCT) that measures the feasibility of the PtDA. A control group (CG) will receive the usual intervention provided by the healthcare centre, while an intervention group (IG) will receive the aid. Participants will be recruited from two different sites of the same institution to distinguish between the CG and the IG. The inclusion criteria involve being a participant older than 18 years of age, having the cognitive ability to provide informed consent and currently deciding whether to continue with active treatment or receive palliative care.

Our pilot study sample size was calculated using the approach proposed by Viechtbauer et al. [44] This method focuses on the ability to identify problems that may arise during the study. Our study will require 59 participants if we want to identify with 95% confidence if a problem exists with a 5% probability in a potential study participant.

To determine if the difference between groups is statistically significant, we will use the *χ* ^2^ test (for qualitative variables), the Student's *t* test or its non‐parametric alternative (for quantitative variables).

Regarding outcome evaluation, a comprehensive plan includes assessing the quality of the decision‐making process and improving decision quality in newly admitted oncology patients [45]. Key indicators such as the level of PtDA utilisation, acquired knowledge, values choice concordance, participation, costs and surrogate health‐related changes will be employed. The diversity of these indicators ensures a comprehensive evaluation covering both the patient's and the healthcare professional's perspectives [28, 45]. To measure knowledge, participants will take a multiple‐choice questionnaire based on the content [45]. Researchers will use the Degner and Sloan Control Preference Scale [28] and the DCS to evaluate the state of uncertainty about which decision to make according to their values and beliefs [46]; we will also use the SDM‐Q‐9 to measure the level of participation on both patients and healthcare professionals. Satisfaction and the quality of care provided to patients will be measured using the Decision Satisfaction Scale [28] and the nine‐item EORTC IN‐PATSAT Scale [24]; the Rini Decision Influence Scale will be used to measure positive influence on the decision [21], while the General Satisfaction Scale [47] will assess professional satisfaction. Finally, the manual by Prades et al. [48] will be used to evaluate the costs of the PtDA considering direct and indirect costs in the implementation of healthcare innovation, which will also be expanded for future cost‐effectiveness evaluations. It is necessary to validate all these scales for the Spanish context.

The post‐implementation protocol establishes evaluations at different time points, from before and after exposure to the PtDA at 1, 6 and 12 months [49]. This approach allows for measuring the evolution of decision‐making and assessing the sustainability of results over time.

We propose various strategies to facilitate sustainability. These include annual training cycles, incentives for the leadership team, periodic updates of the PtDA based on current evidence and an update of the implementation protocol. We recommend updating the PtDA every 2–3 years to reflect the latest evidence (Table 3).

**Table 3:** Timeline of development of a patient decision aid for oncological patients at the end of life (2‐year perspective).

Steps	Tasks	Resources and strategies	Time
Assessment of the context	Make a teamwork	Ottawa Hospital Training + Simulation	1 month
	Authorisation of leaders and managers	Contact the responsible parties	
	Assess local barriers and facilitators for the implementation of DAs	Nonparticipant observation, semi‐structured interviews	
Development of the content and design of the DA	Assess the involvement of professionals and patients in decision‐making	OPTION‐12 scale	3 months
	Development DA	Use the steps of the eight key elements defined by International IPDAS	
	Define content according to the Clinical Practice Guidelines (GPC) on palliative care for adults in end‐of‐life situations	Information to define the content of the video	
	Reach a consensus on the content among professionals, patients and family members	Two separate online meetings will be conducted with the participants.	
	Graphic design of the DA	Design the DAs tailored to the decision to be made using as a reference	
	Conduct alpha and beta pilots	Keys evaluators	3 months
Implementation	Correction and improvements	Utilise the feedback provided by both pilots	
	Action protocol	Description of SDM and its steps. Include specific information on who, when, how and where these DAs should be used.	
	Flowchart action	Develop a flowchart summarising the process to be followed, with the responsible parties, location and timing for decision‐making	
Evaluation and diffusion	Evaluation	Evaluation of the DA: utilisation, knowledge, uncertainty with choices, participation, costs, surrogate health changes and satisfaction of professionals. IPDAS criteria	2 months
	Diffusion	Define communication channels and strategies.	2 weeks
	Following	Conduct periodic evaluation; after the use of DAs, at 1 month, 6 months and 1 year	1 year
	Implementation in other unit care	New patients attending the oncology clinic and requesting a report for palliative care	3 months

Abbreviations: IPDAS, patient decision aid standards; OPTION‐12, Observing Patient Involvement scale; PtDAs, patient decision aids; SDM, shared decision‐making.

## 4 Practice Implications

Implementing PtDA using the JBI framework allows the establishment of evidence‐based practice to improve decision‐making with patients at the end of life.

Careful planning, supported by literature highlighting the benefits of informed decision‐making, promotes a solid foundation for introducing SDM when choosing between invasive treatments or PC. The absence of specific PtDAs for oncology patients in a Spanish setting underscores the need to develop an aid adapted to the local reality. The implementation process relies on resource evaluation, identification of barriers and facilitators and strategies to introduce change. Staff training, particularly for the PC team, is considered essential to enhance SDM skills.

Interdisciplinary collaboration, using computerised reminders and the creation of protocols, is presented as an effective means to integrate the PtDA into clinical practice, optimising resources and facilitating SDM. The continuous evaluation of the process, including alpha and beta pilots, as well as the measurement of multiple indicators, from knowledge to costs and satisfaction, provides a comprehensive perspective on the impact of the PtDA on healthcare. Furthermore, sustainability strategies, such as annual training cycles and periodic evidence‐based updates, ensure the continuity and relevance of the aid over time. This comprehensive approach aims to improve informed decision‐making and healthcare quality for oncology patients at the end of life and establishes a robust and sustainable model for the successful implementation of similar interventions in the future.

## 5 Conclusion

SDM emerges as a crucial component in the clinical setting, particularly for oncology patients in the end‐of‐life stage. The preference for conducting SDM proactively underscores the importance of training healthcare professionals in implementation programmes and the use of PtDAs. This approach has been proven beneficial for patients, enhancing their understanding of treatments and enabling more active participation in the decision‐making process. It is essential that healthcare professionals, as the ones proposing these interventions, are fully informed about the needs and wishes of patients to provide PCC and enhance the quality of healthcare, especially in the EOL, characterised by uncertainty in decision‐making.

## Author Contributions

**Marta Gil Glaría:** conceptualisation; data curation; formal analysis; investigation; methodology; project administration; validation; visualisation; writing–original draft; writing–review and editing. **María Martín Fernández:** software; writing–original draft; writing–review and editing. **Carla Salgado:** writing–original draft; writing–review and editing. **María José Hernández‐Leal:** supervision; conceptualisation; formal analysis; investigation; methodology; project administration; validation; writing–original draft; writing–review and editing.

## Declaration of Generative AI in Scientific Writing

During the preparation of this work, the authors used ChatGPT to improve the transcription language and readability. After using this tool, the authors reviewed and edited the content as needed and now take full responsibility for the content of the publication.

## Ethics Statement

The authors have nothing to report.

## Consent

The authors have nothing to report.

## Conflicts of Interest

The authors declare no conflicts of interest.

## Data Availability

Data sharing is not applicable—no new data generated or the article describes entirely theoretical research.

## References

[1] H. L. Kane , M. T. Halpern , L. B. Squiers , K. A. Treiman , and L. A. McCormack , “Implementing and Evaluating Shared Decision Making in Oncology Practice,” CA: A Cancer Journal for Clinicians 64, no. 6 (2014): 377–388, 10.3322/caac.21245. 25200391

[2] P. J. Barr , I. Scholl , P. Bravo , M. J. Faber , G. Elwyn , and M. McAllister , “Assessment of Patient Empowerment—A Systematic Review of Measures,” PLoS One [Internet] 10, no. 5 (2015): e0126553, 10.1371/journal.pone.0126553. 25970618 PMC4430483

[3] C. Charles , A. Gafni , and T. Whelan , “Self‐Reported Use of Shared Decision‐Making Among Breast Cancer Specialists and Perceived Barriers and Facilitators to Implementing This Approach,” Health Expectations 7, no. 4 (2004): 338–348, 10.1111/j.1369-7625.2004.00299.x. 15544686 PMC5060255

[4] P. Bravo , A. Contreras , L. Perestelo‐Pérez , J. Pérez‐Ramos , and G. Málaga , “En Busca De Una Salud Más Participativa: Compartiendo Decisiones De Salud,” Revista Peruana de Medicina Experimental y Salud Pública [Internet] 30, no. 4 (2014): 691–697, https://rpmesp.ins.gob.pe/index.php/rpmesp/article/view/254/255. 24448951

[5] F. Légaré , S. Ratté , D. Stacey , et al., “Interventions for Improving the Adoption of Shared Decision Making by Healthcare Professionals,” Cochrane Database of Systematic Reviews no. 5 (2010): CD006732, 10.1002/14651858.CD006732.pub2. 20464744

[6] T. H. Wieringa , M. León‐García , N. R. Espinoza Suárez , et al., “The Role of Time in Involving Patients With Cancer in Treatment Decision Making: A Scoping Review,” Patient Education and Counseling 125 (2024): 108285, 10.1016/j.pec.2024.108285. 38701622

[7] C. Munthe , L. Sandman , and D. Cutas , “Person Centred Care and Shared Decision Making: Implications for Ethics, Public Health and Research,” Health Care Analysis 20, no. 3 (2012): 231–249, 10.1007/s10728-011-0183-y. 21786152

[8] S. Pollard , N. Bansback , and S. Bryan , “Physician Attitudes Toward Shared Decision Making: A Systematic Review,” Patient Education and Counseling 98, no. 9 (2015): 1046–1057, 10.1016/j.pec.2015.05.004. 26138158

[9] I. G. Hargraves , V. M. Montori , J. P. Brito , et al., “Purposeful SDM: A Problem‐Based Approach to Caring for Patients With Shared Decision Making,” Patient Education and Counseling 102, no. 10 (2019): 1786–1792, 10.1016/j.pec.2019.07.020. 31353170 PMC6717012

[10] K. Porritt , A. McArthur , C. Lockwood , and Z. Munn , Eds. JBI Handbook for Evidence Implementation (JBI, 2020), https://implementationmanual.jbi.global.

[11] J. Higgins and V. Welch , “Cochrane Handbook for Systematic Reviews of Interventions [Internet].” Cochrane.org, cited December 21, 2023, http://www.training.cochrane.org/handbook.

[12] B. O'Mahony , C. Kerins , C. Murrin , and C. Kelly , “Barriers and Facilitators to the Implementation of Nutrition Standards for School Food: A Mixed‐Methods Systematic Review Protocol,” HRB Open Research 3 (2021): 20, 10.12688/hrbopenres.13041.3. 32743340 PMC7372527

[13] M. J. Page , J. E. McKenzie , P. M. Bossuyt , et al., “The PRISMA 2020 Statement: An Updated Guideline for Reporting Systematic Reviews,” Systematic Reviews 10, no. 1 (2021): 89, 10.1186/s13643-021-01626-4. 33781348 PMC8008539

[14] I. Henselmans , H. W. M. van Laarhoven , P. van Maarschalkerweerd , et al., “Effect of a Skills Training for Oncologists and a Patient Communication Aid on Shared Decision Making About Palliative Systemic Treatment: A Randomized Clinical Trial,” Oncologist 25, no. 3 (2020): e578–e588, 10.1634/theoncologist.2019-0453. 32162796 PMC7066716

[15] D. W. Bos‐van den Hoek , H. W. M. van Laarhoven , R. Ali , et al., “Blended Online Learning for Oncologists to Improve Skills in Shared Decision Making About Palliative Chemotherapy: A Pre‐Posttest Evaluation,” Supportive care in cancer: official journal of the Multinational Association of Supportive Care in Cancer 31, no. 3 (2023): 184, 10.1007/s00520-023-07625-6. 36820944 PMC9947445

[16] D. Mohan , S. C. Alexander , S. K. Garrigues , R. M. Arnold , and A. E. Barnato , “Communication Practices in Physician Decision‐Making for an Unstable Critically Ill Patient With End‐Stage Cancer,” Journal of Palliative Medicine 13, no. 8 (2010): 949–956, 10.1089/jpm.2010.0053. 20642362 PMC4047851

[17] H. Van Veenendaal , L. J. Peters , D. T. Ubbink , et al, “Effectiveness of Individual Feedback and Coaching on Shared Decision‐Making Consultations in Oncology Care: Protocol for a Randomized Clinical Trial,” JMIR Research Protocols [Internet] 11, no. 4 (2022): e35543, 10.2196/35543. 35383572 PMC9021945

[18] L. J. M. Oostendorp , P. B. Ottevanger , A. R. T. Donders , et al., “Decision Aids for Second‐Line Palliative Chemotherapy: A Randomised Phase II Multicentre Trial,” BMC Medical Informatics and Decision Making 17, no. 1 (2017): 130, 10.1186/s12911-017-0529-y. 28859646 PMC5580234

[19] K. V. Dharmarajan , C. B. Walters , T. T. Levin , et al., “A Video Decision Aid Improves Informed Decision Making in Patients With Advanced Cancer Considering Palliative Radiation Therapy,” Journal of Pain and Symptom Management 58, no. 6 (2019): 1048–1055.e2, 10.1016/j.jpainsymman.2019.08.014. 31472276 PMC8132595

[20] R. Agarwal and A. S. Epstein , “Advance Care Planning and End‐of‐Life Decision Making for Patients With Cancer,” Seminars in Oncology Nursing 34, no. 3 (2018): 316–326, 10.1016/j.soncn.2018.06.012. 30100366 PMC6156999

[21] J. N. Dionne‐Odom , R. D. Wells , K. Guastaferro , et al., “An Early Palliative Care Telehealth Coaching Intervention to Enhance Advanced Cancer Family Caregivers' Decision Support Skills: The CASCADE Pilot Factorial Trial,” Journal of Pain and Symptom Management [Internet] 63, no. 1 (2022): 11–22, 10.1016/j.jpainsymman.2021.07.023. 34343621 PMC8881798

[22] L. Fritz , H. Zwinkels , J. A. F. Koekkoek , et al., “Advance Care Planning in Glioblastoma Patients: Development of a Disease‐Specific ACP Program,” Supportive Care in Cancer 28, no. 3 (2020): 1315–1324, 10.1007/s00520-019-04916-9. 31243585 PMC6989589

[23] M. Hoerger , R. M. Epstein , P. C. Winters , et al., “Values and Options in Cancer Care (VOICE): Study Design and Rationale for a Patient‐Centered Communication and Decision‐Making Intervention for Physicians, Patients With Advanced Cancer, and Their Caregivers,” BMC Cancer 13, no. 1 (2013): 188, 10.1186/1471-2407-13-188. 23570278 PMC3637237

[24] I. J. Korfage , G. Carreras , C. M. Arnfeldt Christensen , et al., “Advance Care Planning in Patients With Advanced Cancer: A 6‐Country, Cluster‐Randomised Clinical Trial,” PLoS Medicine 17, no. 11 (2020): e1003422, 10.1371/journal.pmed.1003422. 33186365 PMC7665676

[25] N. Michael , C. O'callaghan , E. Georgousopoulou , A. Melia , M. Sulistio , and D. Kissane , “Video Decision Support Tool Promoting Values Conversations in Advanced Care Planning in Cancer: Protocol of a Randomised Controlled Trial,” BMC palliative care 20, no. 1 (2021): 95, 10.1186/s12904-021-00794-3. 34167538 PMC8229383

[26] J. A. Rietjens , I. J. Korfage , L. Dunleavy , et al., “Advance Care Planning—A Multi‐Centre Cluster Randomised Clinical Trial: The Research Protocol of the ACTION Study,” BMC Cancer 16, no. 1 (2016): 264, 10.1186/s12885-016-2298-x. 27059593 PMC4826555

[27] S. T. Tang , J.‐S. Chen , F.‐H. Wen , et al., “Advance Care Planning Improves Psychological Symptoms but not Quality of Life and Preferred End‐Of‐Life Care of Patients With Cancer,” Journal of the National Comprehensive Cancer Network 17, no. 4 (2019): 311–320, 10.6004/jnccn.2018.7106. 30959470

[28] C. Tricou , S. Yennu , M. Ruer , E. Bruera , and M. Filbet , “Decisional Control Preferences of Patients With Advanced Cancer Receiving Palliative Care,” Palliative and Supportive Care 16, no. 5 (2018): 544–551, 10.1017/s1478951517000803. 29094668

[29] L. Perestelo‐Pérez , J. Pérez‐Ramos , A. Rivero‐Santana , D. Carballo‐González , and P. Serrano‐Aguilar (coord.) y Grupo de Trabajo del manual metodológico para evaluar la calidad de las HATD . *Manual con criterios de evaluación y validación de las Herramientas de Ayuda para la Toma de Decisiones*. Línea de desarrollos metodológicos de la Red Española de Agencias de Evaluación de Tecnologías y Prestaciones del SNS (Madrid: Ministerio de Sanidad, Servicios Sociales e Igualdad. Servicio de Evaluación del Servicio Canario de la Salud, 2013).

[30] Grupo de trabajo de la Guía de Práctica Clínica sobre atención paliativa al adulto en situación de últimos días. *Guía de Práctica Clínica sobre atención paliativa al adulto en situación de últimos días. Guías de Práctica Clínica en el SNS* (Madrid: Ministerio de Sanidad; Santiago de Compostela: Agencia de Conocimiento en Salud (ACIS). Unidad de Asesoramiento Científico‐técnico, Avalia‐t, 2021).

[31] H. Otani , T. Morita , T. Esaki , et al., “Burden on Oncologists When Communicating the Discontinuation of Anticancer Treatment,” Japanese Journal of Clinical Oncology 41, no. 8 (2011): 999–1006, 10.1093/jjco/hyr092. 21764830 PMC3146312

[32] J. C. Bermejo Higuera , B. Lozano González , M. Villacieros Durbán , and M. Gil Vela , “Atención Espiritual En Cuidados Paliativos Valoración y Vivencia de Los Usuarios,” Medicina Paliativa 20, no. 3 (2013): 93–102, 10.1016/j.medipa.2012.05.00.

[33] G. Phillips , K. Lifford , A. Edwards , M. Poolman , and N. Joseph‐Williams , “Do Published Patient Decision Aids for End‐of‐Life Care Address Patients' Decision‐Making Needs? A Systematic Review and Critical Appraisal,” Palliative Medicine 33, no. 8 (2019): 985–1002, Available at: 10.1177/0269216319854186. 31199197

[34] P. A. Harris , R. Taylor , B. L. Minor , et al, “The REDCap Consortium: Building an International Community of Software Platform Partners,” Journal of Biomedical Informatics [Internet] 95, no. 103208 (2019): 103208, 10.1016/j.jbi.2019.103208. 31078660 PMC7254481

[35] F. Légaré , A. M. O'Connor , I. D. Graham , et al., “Primary Health Care Professionals' Views on Barriers and Facilitators to the Implementation of the Ottawa Decision Support Framework in Practice,” Patient Education and Counseling 63, no. 3 (2006): 380–390, 10.1016/j.pec.2006.04.011. 17010555

[36] M. Ortega‐Moreno , N. Padilla‐Garrido , L. Huelva‐López , F. Aguado‐Correa , J. Bayo‐Calero , and E. Bayo‐Lozano , “Barreras Y Facilitadores Para La Implementación De La Toma De Decisiones Compartidas En Oncología: Percepciones De Los Pacientes,” Revista de Calidad Asistencial 32, no. 3 (2017): 141–145, 10.1016/j.cali.2017.01.002. 28274548

[37] F. Légaré , S. Ratté , K. Gravel , and I. D. Graham , “Barriers and Facilitators to Implementing Shared Decision‐Making in Clinical Practice: Update of a Systematic Review of Health Professionals’ Perceptions,” Patient Education and Counseling 73, no. 3 (2008): 526–535, 10.1016/j.pec.2008.07.018. 18752915

[38] A. M. Botella Nicolás and P. Ramos Ramos , “Investigación‐Acción Y Aprendizaje Basado En Proyectos,” Perfiles Educativos 41, no. 163 (2019): 109–122, 10.22201/iisue.24486167e.2019.163.58923.

[39] Ottawa Hospital Research Institute & University of Ottawa . “Decision Coaching [Internet],” Ohri.ca (2024), https://decisionaid.ohri.ca/coaching.html.

[40] Ohri.ca, “Guía de Apoyo Decisional de Ottawa [Internet],” cited December 21, 2023, https://decisionaid.ohri.ca/docs/das/GADO.pdf.

[41] Ottawa Hospital Research Institute and University of Ottawa. “Guía de Apoyo Decisional de Ottawa [Internet]” (2012), http://decisionaid.ohri.ca/opdg_video.html.

[42] D. V. Orjuela and M. H. Osses , “Percepción De La Simulación Clínica Como Estrategia De Enseñanza Para El Desarrollo De Competencias Transversales En Terapia Ocupacional,” Cadernos Brasileiros de Terapia Ocupacional [Internet] 29 (2021): e2910, 10.1590/2526-8910.ctoao2199.

[43] T. Walsh , P. J. Barr , R. Thompson , E. Ozanne , C. O'neill , and G. Elwyn , “Undetermined Impact of Patient Decision Support Interventions on Healthcare Costs and Savings: Systematic Review,” BMJ (Clinical Research ed.) 348 (2014): g188, 10.1136/bmj.g188. PMC3900320 24458654

[44] W. Viechtbauer , L. Smits , D. Kotz , et al., “A Simple Formula for the Calculation of Sample Size in Pilot Studies,” Journal of Clinical Epidemiology 68, no. 11 (2015): 1375–1379, https://www.jclinepi.com/article/S0895-4356(15)00303-0/abstract. 26146089 10.1016/j.jclinepi.2015.04.014

[45] K. R. Sepucha and I. Scholl , “Measuring Shared Decision Making: A Review of Constructs, Measures, and Opportunities for Cardiovascular Care,” Circulation: Cardiovascular Quality and Outcomes 7, no. 4 (2014): 620–626, 10.1161/CIRCOUTCOMES.113.000350. 24867916

[46] M. Urrutia , S. Campos , and A. O'Connor , “Validation of a Spanish Version of the Decisional Conflict Scale,” Revista Medica de Chile 136, no. 11 (2008): 1439–1447, 10.4067/s0034-98872008001100010. 19301775

[47] C. Carrillo‐García , M. E. Martínez‐Roche , C. I. Gómez‐García , and M. Meseguer‐DePedro , “Satisfacción Laboral De Los Profesionales Sanitarios De Un Hospital Universitario: Análisis General Y Categorías Laborales,” Anales de Psicología 31, no. 2 (2015): 645, 10.6018/analesps.31.2.169791.

[48] J. Pinto‐Prades , J. Sánchez Martínez , and J. Abellán Perpiñán , Métodos Para la Evaluación Económica de Nuevas Prestaciones (Ministerio de Sanidad y Consumo, 2003), https://www.sanidad.gob.es/estadEstudios/estadisticas/docs/metodos_evaluacion.pdf.

[49] G. Elwyn and T. Miron‐Shatz , “Deliberation Before Determination: The Definition and Evaluation of Good Decision Making,” Health Expectations 13, no. 2 (2010): 139–147, 10.1111/j.1369-7625.2009.00572.x. 19740089 PMC5060530

